# Prevalence of Depression and Anxiety in Cancer Patients Receiving Palliative Care in the Comprehensive Cancer Center, King Fahad Medical City, Riyadh, Saudi Arabia: A Cross-Sectional Study

**DOI:** 10.7759/cureus.83493

**Published:** 2025-05-05

**Authors:** Nawaf A Alotaibi, Abdullah I Alsuhail

**Affiliations:** 1 Palliative Care, Comprehensive Cancer Center, King Fahad Medical City, Riyadh, SAU

**Keywords:** anxiety, depression, hads, palliative care, saudi arabia

## Abstract

Background

Depression and anxiety are common psychological challenges among cancer patients, particularly those receiving palliative care. These conditions significantly impact the quality of life but are often underdiagnosed and undertreated. This study evaluates depression and anxiety prevalence in Saudi palliative patients using the Arabic Hospital Anxiety and Depression Scale (HADS), identifies psychological distress factors, and proposes clinical recommendations to improve regional mental health screening protocols in palliative patients.

Methods

A cross-sectional study was conducted with 130 palliative care patients at King Fahad Medical City, Riyadh. The Arabic version of the Hospital Anxiety and Depression Scale (HADS) was used to assess depression and anxiety levels. Data were analyzed using the Statistical Package for Social Sciences (SPSS) (IBM Corp., Armonk, NY), with a significance level of p<0.05.

Results

Sixty-five participants (50%) had HADS for depression (HADS-D) scores of ≥8 (indicating depression), while 40 (31%) had HADS for anxiety (HADS-A) scores of ≥8 (indicating anxiety). Women exhibited higher levels of depression and anxiety compared to men, though this difference was not statistically significant. Pain and fatigue were strongly associated with higher rates of depression (p=0.014 and p=0.002, respectively). Patients with metastatic disease showed lower levels of anxiety (p=0.011). Patients taking antidepressants had significantly higher rates of depression and anxiety (p=0.024).

Conclusion

The high prevalence of depression and anxiety among cancer patients receiving palliative care emphasizes the importance of implementing a routine psychological screening in oncology settings. The study findings recommend that individuals exhibiting persistent and severe physical symptoms be assessed for possible mood disorders. Contrary to expectations, metastatic patients reported lower anxiety levels. However, this warrants further study to disentangle the roles of coping mechanisms, palliative interventions, and cultural factors.

## Introduction

Depression and anxiety represent prevalent psychological comorbidities among individuals diagnosed with cancer and those receiving palliative care, posing significant challenges to their overall well-being and quality of life [1]. The intricate interplay between the physical manifestations of cancer and the psychological burden of emotional distress underscores the multifaceted nature of care required for patients facing these complex conditions [2].

Mood disorders rank high among the most prevalent and impactful psychiatric conditions. Depression is known to coincide with various physical manifestations in individuals with advanced cancer, occurring in approximately 29% of cases [3,4]. Studies have shown that mood disorders in medically ill patients are frequently overlooked, leading to inadequate treatment [5]. Even terminally ill patients experiencing depression could derive benefits from intervention, even during the final stages of life [6]. Understanding the nuances of depression in cancer patients is essential for healthcare providers to deliver comprehensive, patient-centered care that addresses not only the physical aspects of cancer but also the emotional and mental health needs of individuals grappling with the disease [5].

Screening for depression in oncology patients is challenging. Screening tools are valuable in identifying depression symptoms in cancer patients, enabling healthcare providers to initiate appropriate interventions and support tailored to the individual's mental health needs [7]. The Hospital Anxiety and Depression Scale (HADS) is a self-assessment questionnaire designed to measure levels of anxiety and depression in individuals, particularly patients with medical conditions such as cancer. The HADS consists of 14 items divided into two subscales: HADS for anxiety (HADS-A) and HADS for depression (HADS-D) [8]. In a study assessing the effectiveness of the Hospital Anxiety and Depression Scale (HADS), the WHO (five) Well Being Index (WBI-5), and the Patient Health Questionnaire (PHQ) for depression screening, all three questionnaires exhibited proficient performance in detecting symptoms of depression and anxiety [9]. The HADS evaluates emotional distress on a broader scale while providing the advantage of excluding somatic items [5].

An official validation study has rigorously examined and endorsed the Arabic adaptation of the Hospital Anxiety and Depression Scale (HADS), affirming its credibility and precision in evaluating anxiety and depression levels within the Arabic-speaking population [10]. This study aims to provide a systematic evaluation of depression and anxiety prevalence among palliative care patients in Saudi Arabia using validated Arabic HADS instruments, addressing critical gaps in regional mental health data in this population. By identifying key sociodemographic and clinical factors associated with psychological distress, the findings will provide empirically grounded recommendations for optimizing mental health screening protocols and support services within Saudi Arabia's developing palliative care infrastructure.

## Materials and methods

Study design and participants

This cross-sectional study obtained approval from the Institutional Review Board (IRB) of King Fahad Medical City (approval number: 23-632) to investigate the prevalence of depression and anxiety among patients benefiting from palliative care services. The study enrolled palliative patients who were recipients of palliative services, adults (≥18 years old), diagnosed with cancer, and Arabic-speaking. The research excluded individuals under 18 years of age and those who were cognitively or consciously impaired to ensure valid data collection.

Participants

The participants were randomly selected from the patient list of the Comprehensive Cancer Center at King Fahad Medical City. The sample consisted of individuals who were able to provide informed consent and complete the study questionnaire.

Data collection

Data collection was conducted utilizing a questionnaire administered through Google Forms (Google, Inc., Mountain View, CA). The survey included questions regarding the patient's age, gender, type of cancer, time since diagnosis, metastasis status, current treatment regimen, ongoing antidepressant use, "do not resuscitate" (DNR) status, and recorded Palliative Performance Scale (PPS) score.

Assessment tools

To evaluate levels of depression and anxiety, we used the Arabic version of HADS, validated by Terkawi et al. (2017) in a large Arabic-speaking clinical population. This adaptation demonstrated high internal consistency (α>0.85 for both subscales), preserved the original two-factor structure, and showed strong concordance with other mental health measures (e.g., Depression Anxiety Stress Scale-21 {DASS-21}) [10]. This standardized instrument consists of two subscales: HADS-D for depression and HADS-A for anxiety, each with seven questions equating to a total score of 21 points. Scoring interpretation included 0-7 points for normal, 8-10 for borderline abnormal (borderline case), and 11-21 for abnormal case. In our study, patients with HADS scores of ≥8 were considered to have depression or anxiety [8,11].

Statistical analysis

The collected data were analyzed using the Statistical Package for Social Sciences (SPSS) (IBM Corp., Armonk, NY). A significance level of less than 0.05 was chosen for all statistical tests to determine the presence of significant results. Chi-square tests were used to explore the associations between categorical variables and the anxiety and depression scores. Additionally, correlation analyses were conducted to assess how variables correlate with anxiety and depression scores.

## Results

**Figure 1:**
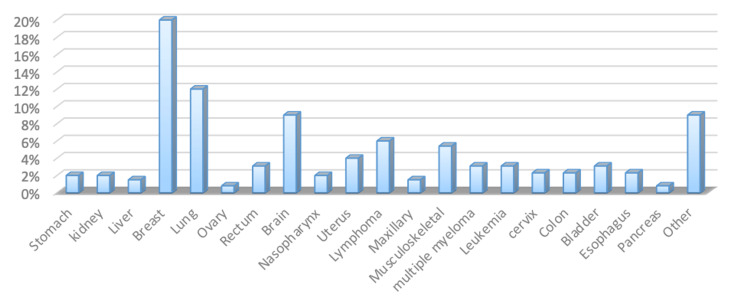
Participants' primary diagnosed cancer

**Figure 2:**
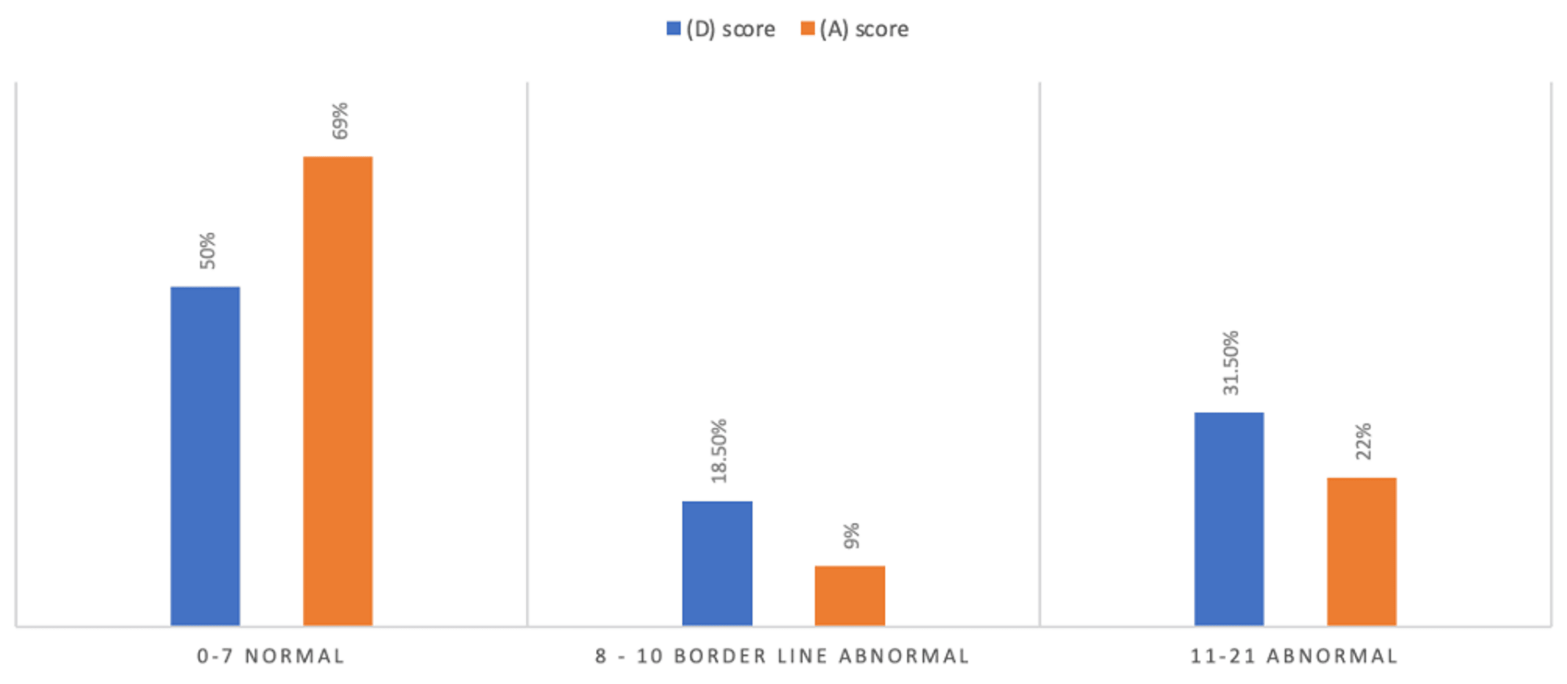
Participants' HADS scoring HADS: Hospital Anxiety and Depression Scale

The study encompassed 130 participants, with a gender distribution of 43 men (33%) and 87 women (67%). Among the cancer patients, the majority, 80 individuals (61%), were aged over 50 years, as shown in Table 1. Notably, breast cancer was identified as the most prevalent cancer type, accounting for 27 cases (21%), followed by lung cancer at 16 cases (12%) and brain cancer at 11 cases (9%), as demonstrated in Figure 1. The findings show that among the participants, 65 (50%) scored between 0 and 7 on the HADS-D scale, 24 (18.5%) scored between 8 and 10, and 41 (31.5%) scored between 11 and 21. Regarding the HADS-A scale, 90 (69%) scored between 0 and 7, 12 (9%) scored between 8 and 10, and 28 (22%) scored between 11 and 21 (Figure 2). Furthermore, 65 (50%) of the participants had HADS-D scores of ≥8, while 40 (31%) had HADS-A scores of ≥8. In our study, patients with HADS scores of ≥8 were considered to have depression or anxiety.

**Table 1:** Participant characteristics (n=130)

Characteristics	Number of patients (n=130)	Percentage (%)
Gender		
Male	43	33%
Female	87	67%
Age		
<30	8	6%
30-39	14	11%
40-49	28	22%
50-59	35	27%
≥60	45	34%
Marital status		
Single	15	12%
Married	71	55%
Divorce	11	9%
Widow	33	24%
Time since first diagnosis		
<1 year	24	19%
1-5 years	78	60%
6-10 years	28	21%
Patient using antidepressant		
Yes	26	20%
No	104	80%
Patient who has metastatic disease		
Yes	61	47%
No	69	53%

The data revealed no significant association between any type of cancer and depression or anxiety. Patients on antidepressants exhibited significantly higher rates of depression and anxiety with HADS scores of ≥8 (p-value: 0.024). Additionally, an inverse correlation was observed between patients with metastatic disease and anxiety with HADS-A scores of ≥8, with a statistically significant p-value of 0.011 and a correlation coefficient of r=-0.223 (Table 2), as confirmed through chi-square testing that patients with metastatic disease exhibit lower levels of anxiety, with a p-value of 0.022 (Table 3). Women exhibited higher levels of depression and anxiety compared to men with HADS-A scores of ≥8, although it is not statistically significant. Notable positive correlations were noticed between patients diagnosed with cancer for more than five years and depression (p-value: 0.049).

**Table 2:** Correlation between depression and anxiety with participants' variables PPS, Palliative Performance Scale; HADS, Hospital Anxiety and Depression Scale; HADS-D, HADS for depression; HADS-A, HADS for anxiety

	HADS-D		HADS-A	
	Correlation, r	P-value	Correlation, r	P-value
Age	-0.082	0.35	-0.105	0.234
Gender	0.035	0.69	0.064	0.47
Marital status	0.004	0.967	0.064	0.471
Time since first diagnosis	0.173	0.049	0.135	0.126
Primary cancer	0.042	0.634	0.081	0.362
Patient using antidepressant	0.248	0.004	0.358	0.001
Patient who has metastatic disease	-0.135	0.126	-0.223	0.011
PPS score	-0.202	0.02	-0.067	0.45
Pain	0.215	0.014	0.149	0.09
Tiredness	0.265	0.002	0.133	0.132
Drowsiness	0.140	0.112	0.172	0.051
Nausea	0.091	0.3	0.155	0.08
Lack of appetite	0.187	0.03	0.178	0.04
Shortness of breath	-0.018	0.836	-0.074	0.403

**Table 3:** Participants' variables and their association with depression and anxiety using the chi-square test PPS, Palliative Performance Scale; HADS, Hospital Anxiety and Depression Scale; HADS-D, HADS for depression; HADS-A, HADS for anxiety

	HADS-D score		P-value	HADS-A score		P-value
	Normal<8	Abnormal≥8		Normal<8	Abnormal≥8	
Gender						
Male	22 (51%)	21 (49%)	0.5	32 (74%)	11 (26%)	0.244
Female	43 (49%)	44 (51%)		58 (67%)	29 (33%)	
Age						
<30	3 (38%)	5 (62%)	0.752	5 (63%)	3 (37%)	0.817
30-39	7 (50%)	7 (50%)		8 (57%)	6 (43%)	
40-49	15 (54%)	13 (46%)		20 (71%)	8 (29%)	
50-59	15 (43%)	20 (57%)		24 (69%)	11 (31%)	
≥60	25 (56%)	20 (44%)		33 (73%)	12 (27%)	
Marital status						
Single	6 (40%)	9 (60%)	0.838	10 (67%)	5 (33%)	0.453
Married	37 (52%)	34 (48%)		53 (75%)	18 (25%)	
Divorce	6 (55%)	5 (45%)		6 (55%)	5 (45%)	
Widow	16 (48%)	17 (52%)		21 (64%)	12 (36%)	
Time since first diagnosis						
<1 year	14 (58%)	10 (42%)	0.45	19 (79%)	5 (21%)	0.277
1-5 years	40 (51%)	38 (49%)		54 (69%)	24 (31%)	
6-10 years	11 (39%)	17 (61%)		17 (61%)	11 (39%)	
Using antidepressant						
No	57 (55%)	47 (45%)	0.024	81 (78%)	23 (22%)	0.001
Yes	8 (31%)	18 (69%)		9 (35%)	17 (65%)	
Metastatic disease						
No	31 (45%)	38 (55%)	0.146	42 (61%)	27 (39%)	0.022
Yes	34 (56%)	27 (44%)		48 (79%)	13 (21%)	
PPS score						
30%	2 (29%)	5 (71%)	0.159	4 (57%)	3 (43%)	0.496
40%-50%	8 (33%)	16 (67%)		14 (58%)	10 (42%)	
60%-70%	39 (56%)	31 (44%)		51 (73%)	19 (27%)	
80%-90%	16 (55%)	13 (45%)		21 (72%)	8 (28%)	

The data further revealed that patients with lower Palliative Performance Scale (PPS) scores had a heightened prevalence of depression, supported by a correlation coefficient of r=-0.202 and p-value of 0.021. Contrary, Table 3 illustrates no significant relationship between lower PPS scores and depression, with a p-value of 0.16. Moreover, patients reporting pain and fatigue exhibited significantly increased rates of depression compared to others (p-values of 0.014 and 0.002, respectively). Furthermore, pain was significantly associated with anxiety (p-value: 0.026) but without a significant correlation (p-value: 0.092).

## Discussion

This study showed that depression and anxiety are prevalent in advanced cancer patients, impacting their mental well-being and quality of life [12]. In our study, it was identified that 65 (50%) of the participants had HADS-D scores of ≥8, while 40 (31%) scored ≥8 on the HADS-A scale. Women exhibited a higher prevalence of depression and anxiety. Significant associations were observed between pain and the occurrence of both depression and anxiety.

A systematic review and meta-analysis highlighted the prevalence of depression among cancer patients in palliative care, with the highest rates observed in Africa at 36% and the lowest in Europe at 25% [13]. Smith et al.'s study in Manchester, England, reported 25% experiencing anxiety and 22% with depression [1]. In a study in China, around 29% and 11% of patients exhibited anxiety and depression, respectively [14]. A study by Azevedo et al. in Brazil, involving 115 palliative care patients at primary healthcare, revealed a 42% depression rate [15]. In contrast, a study conducted by Gontijo Garcia et al. in a hospital setting utilizing the HADS score found that 44% of the participants had depression with HADS-D scores of ≥8, while 25.7% experienced anxiety with HADS-A scores of ≥8 [16].

In our study, patients with pain, fatigue, and a lack of appetite showed a higher propensity to have depression. A study conducted in Houston, USA, found that patients with depressive moods tended to demonstrate significantly frequent occurrences of symptoms such as drowsiness, nausea, pain, dyspnea, and decreased appetite and challenges to their overall well-being [2]. The occurrence of cancer pain is correlated with a 3.5 times higher risk of experiencing depression and a 2.5 times higher likelihood of feeling anxious [17,18]. Additionally, depression is associated with more than double the risk of hospitalization for opioid poisoning and opioid abuse or dependence in patients with cancer pain [19]. A study in India, involving 274 cancer patients divided into intervention and usual-care groups, demonstrated that better management of depression leads to improvements in pain over a 12-month period [20].

In Saudi Arabia, various studies have investigated the prevalence of anxiety and depression across different cancer patient populations. A study focusing on patients with colorectal cancer reported a 30.0% rate of depression identified using the Structured Clinical Interview for Depression (SCID) [21]. Additionally, research on breast cancer patients applied the Hospital Anxiety and Depression Scale (HADS), highlighting that 57% exhibited moderate to severe depression while 44% showed moderate to severe anxiety [22]. In the southern region of Saudi Arabia, a study on cancer patients reported prevalence rates of depression and anxiety at 42.4% and 23.9%, respectively [23]. Furthermore, a study involving patients with hematological malignancies utilized the Patient Health Questionnaire-9 and Generalized Anxiety Disorder-7 (GAD-7), revealing depression in 46.5% and anxiety in 22.3% [24]. Another research using the Depression Anxiety Stress Scale (DASS) indicated high rates of depression (44.8%), anxiety (52.5%), and stress (42.7%) [25]. In Saudi Arabia in 2022, a national screening program of the general population revealed the prevalence of major depressive disorder (MDD) and Generalized Anxiety Disorder (GAD) to be 12.7% and 12.4%, respectively [26].

In our study, patients who were on antidepressants demonstrated a higher level of depression and anxiety (p-value: 0.024). We attributed this finding to the preexisting diagnoses of depression and anxiety in these individuals, leading to the initiation of medication. In a randomized clinical trial, the prophylactic administration of an antidepressant (citalopram) to prevent depression in head and neck cancer patients resulted in significantly reduced depression levels in the treatment group compared to the control group receiving a placebo, after a 12-week intervention period [27].

This study has several important limitations to consider. First, while our sample size (N=130) is comparable to similar psycho-oncology studies, it restricts the precision of subgroup analyses (particularly for specific antidepressant regimens) and may obscure smaller but clinically meaningful effects [1,2]. Second, the single-center design and consecutive enrollment, though methodologically sound, may limit the demographic diversity and generalizability of findings to other healthcare settings or cultural contexts. Third, as a cross-sectional study, we cannot establish temporal relationships or account for all potential confounders such as socioeconomic status, social support systems, or detailed mental health history. These limitations highlight the need for future multicenter longitudinal studies with larger, more diverse samples to confirm and extend our findings.

## Conclusions

The high prevalence of depression and anxiety among cancer patients receiving palliative care emphasizes the importance of implementing a routine psychological screening in palliative patients. The study findings recommend that individuals exhibiting persistent and severe physical symptoms be assessed for possible mood disorders. Contrary to expectations, metastatic patients reported lower anxiety levels. However, this warrants further study to disentangle the roles of coping mechanisms, palliative interventions, and cultural factors. We recommend future multicenter longitudinal studies with larger, more diverse samples to confirm and extend our findings.
